# Tin Tumbler Taken from a Patient, Post-mortem, and Which Had Been Introduced by Him into the Rectum, for the Purpose of Reducing Prolapsus of That Intestine

**Published:** 1855-09

**Authors:** 


*Tin Tumbler taken from a patient, post-mortem, and which had been introduced by him into the rectum, for the purpose of reducing prolapsus of that intestine.*—The tumbler was sent to the Boston Society for Medical Improvement by Dr. John O. Stone, of New York city, and was presented, and the account of the case read, by Dr. H. O. Stone, of Boston. The following is an abstract of the detailed description of the case, read by Dr. S., and which was published in the Boston Medical and Surgical Journal of May 14th, 1834.

The patient introduced the tumbler on the 4th of April, 1834, causing its entrance into the bowel by sitting upon it. The tumbler being drawn upwards with the returning intestine, attempts were made by the patient to extract it, with his fingers, and by means of "shoe-maker's forceps." "With these he had considerably broken and flattened the edge of the base or rim, of the tumbler, and forced it beyond the rectum, into the colon." It was found in this situation by the physician who was summoned, Dr. G. Moodie, of North Andover, Mass. Dr. M. introduced his "hand and arm into the rectum, seized" the tumbler and "made a powerful" but unsuccessful "effort to extract it." The blunt hook was next tried, without extracting the tumbler, although it was brought down so that "it could be seen." "Owing to its flattened state, it hitched in the plicæ of the intestine." Several physicians and surgeons were called in consultation; among others, Dr. Joseph Kittredge, of Andover, and Dr. Whiting, of Haverhill. No efforts at extraction by the hook or the fingers were of any avail; although the tumbler was brought into view and seized, powerful efforts being again made to disengage it from its situation. One of the practitioners again introduced his hand, but could not bring the tumbler away. The patient asked to have his abdomen opened, and the foreign body thus removed. "He was told that this would produce certain death." A proposition to divide the levatores ani was negatived by Dr. Kittredge, who feared fatal hæmorrhage. "The patient lived about three days after this. His tongue sloughed, and there was gangrene of the large intestines. The tumbler was extracted after death: it measured 3½ inches in length, 3½ inches in width, in the direction of the flattened part, and 2 inches across its base; it would hold nearly three gills." It is preserved in the Society's Cabinet.—*Boston Medical and Surgical Journal.*

